# Diversity and population structure of Nordic potato cultivars and breeding clones

**DOI:** 10.1186/s12870-022-03726-2

**Published:** 2022-07-18

**Authors:** Catja Selga, Pawel Chrominski, Ulrika Carlson-Nilsson, Mariette Andersson, Aakash Chawade, Rodomiro Ortiz

**Affiliations:** 1 Department of Plant Breeding, Swedish University of Agricultural Sciences (SLU), Alnarp, Sweden; 2 Nordiskt Genresurscenter (NordGen), Alnarp, Sweden

**Keywords:** Genebank, Genetic diversity, Population structure, Potato, Potato breeding

## Abstract

**Background:**

The genetic diversity and population structure of breeding germplasm is central knowledge for crop improvement. To gain insight into the genetic potential of the germplasm used for potato breeding in a Nordic breeding program as well as all available accessions from the Nordic genebank (NordGen), 133 potato genotypes were genotyped using the Infinium Illumina 20 K SNP array. After SNP filtering, 11 610 polymorphic SNPs were included in the analysis. In addition, data from three important breeding traits – percent dry matter and uniformity of tuber shape and eye – were scored to measure the variation potato cultivars and breeding clones.

**Results:**

The genetic diversity among the genotypes was estimated using principal coordinate analysis based on the genetic distance between individuals, as well as by using the software STRUCTURE. Both methods suggest that the collected breeding material and the germplasm from the gene-bank are closely related, with a low degree of population structure between the groups. The phenotypic distribution among the genotypes revealed significant differences, especially between farmer’s cultivars and released cultivars and breeding clones. The percent heterozygosity was similar between the groups, with a mean average of 58–60%. Overall, the breeding germplasm and the accessions from the Nordic genebank seems to be closely related with similar genetic background.

**Conclusion:**

The genetic potential of available Nordic potato breeding germplasm is low, and for genetic hybridization purposes, genotypes from outside the Nordic region should be employed.

**Supplementary Information:**

The online version contains supplementary material available at 10.1186/s12870-022-03726-2.

## Introduction

The cultivated potato (*Solanum tuberosum* L.) originates from the Andes in South America, where it was domesticated 8 000–10 000 years ago [51]. The import of potato landraces, particularly from southern Chile to northern latitudes in Europe, resulted in potato cultivars already adapted to long-days. This adaptation was later found to be very favourable for cultivation in Europe and contributed to the genetic background of many popular cultivars grown today [17, 20, 21]. The potato was first introduced in Europe in the second half of the sixteenth century [22] and from early on, several different genotypes were brought to Europe from South America [46]. Over the course of the seventeenth and eighteenth centuries, potato gained acceptance as a food crop in Europe [2], and became particularly popular in northern Europe [31].

Potato reached the Nordic region of Europe (Sweden, Denmark, Norway, Finland, and Iceland) later than the rest of the countries in Europe. It was introduced to Sweden in 1658; i.e., about 50 years after other European sites [8]. The first potato plants cultivated in the Nordic region originated from several European sites [5, 12, 36, 53, 55]. These first plants established the genetic background to the farmer’s cultivars (sometimes referred to as landraces [30]), which were popular before professional potato breeding began in the early twentieth century [12] and often resulted in the sowing of potato true seeds rather than tubers in early days of potato cultivation [39].

Sweden was the first country in the Nordic region to start a formal potato breeding program through work at the Swedish Seed Association (Svalöf) in 1903 [11, 38]. This breeding program changed ownership throughout the twentieth century and is since 2006 situated at the Department of Plant Breeding at the Swedish University of Agricultural Sciences (SLU).

Since 1979, the Nordic Genetic Resource Center (NordGen) has collected released potato cultivars, old farmer’s cultivars, and breeding clones from the Nordic countries for future conservation [56]. The potato accessions at NordGen are kept *in vitro*, and metadata such as morphological characteristics, collecting site, release year, and pedigree information are kept in their database GENBIS. Their collection of farmer’s cultivars was characterised by Veteläinen et al. [56] studying a wide range of morphological traits and by using 63 amplified fragment length polymorphism (AFLP) markers. In this study, no population structure based on country origin could be revealed either by studying the phenotypic or genotypic data. After the study was published, new tools and methods have made high-throughput genotypic data more accessible and affordable with the release of the potato genome reference sequence [43].

In our research that we report herein we studied the diversity of 133 potato genotypes from SLU and NordGen. Genotypic data was collected using the GGPv3.0 array [57], and the single nucleotide polymorphism (SNP) data collected were used to evaluate the genetic structure and level of heterozygosity within each and among the three populations. Phenotypic data were also recorded for three important breeding traits as a measure of the variation between the groups.

## Results

### Phenotypic variation

The sample of genotypes included in this study is of the table potato type grown in the Nordic region of Europe – particularly Sweden. The genotypes were grouped based on two criteria 1) source of the germplasm (i.e. cultivars grown today, Nordic Genetic Resources Center [NordGen] and, Swedish University of Agricultural Sciences [SLU]), or 2) clonal type (i.e. breeding clones, released cultivars and farmer’s cultivars). Phenotypic characteristics of importance to potato marketing – percent dry matter in the tuber flesh, tuber eye depth and tuber shape uniformity are shown in Table 1. Variation among the three sources of data (NordGen, SLU and cultivar grown today) was observed and a significant (*P* < 0.001) difference was found for percent dry matter and tuber shape uniformity. According to Tukey’s range test the accessions from NordGen displayed, on average, significantly different percent dry matter and tuber shape uniformity than the two other groups (SLU and cultivar grown today). An alternative division of genotypes was by grouping them by clonal type – breeding clones, released cultivars or farmer’s cultivar. There were significant differences for all measured phenotypic traits –percent dry matter, tuber eye depth, and tuber shape uniformity among these three groups (*P* < 0.001) (Table 2). For percent dry matter and tuber eye depth, Tukey’s range test presented differences between farmer’s cultivars and the other two groups. For tuber shape uniformity, Tukey’s range test presented differences between breeding clones and the two other groups.

**Table 1:** Means and variances for percent tuber dry matter, tuber eye depth and uniformity of tuber shape in breeding clones, accessions held by NordGen and cultivars grown today by Nordic farmers using a scale ranging from 1 (non-uniform) to 9 (uniform) for tuber shape uniformity, from 1 (deep) to 9 (shallow) for tuber eye depth, and 1 (low) to 9 (high) for percent dry matter content in tubers

	Mean	Variance
Percent tuber dry matter		
Breeding clones	4.86	4.88
NordGen accessions	6.30	3.42
Cultivars grown today	4.24	0.97
Tuber eye depth		
Breeding clones	6.06	0.83
NordGen accessions	5.62	2.36
Cultivars grown today	5.87	0.54
Uniformity of tuber shape		
Breeding clones	5.15	2.20
NordGen accessions	4.53	2.82
Cultivars grown today	5.11	0.78

**Table 2:** Mean and variance for percent tuber dry matter, tuber eye depth and uniformity of tuber shape for breeding clones and cultivars using a scale ranging from 1 (non-uniform) to 9 (uniform) for tuber shape uniformity, from 1 (deep) to 9 (shallow) for tuber eye depth, and 1 (low) to 9 (high) for percent dry matter content in tubers

	Mean	Variance
Percent tuber dry matter		
Breeding clones	5.03	3.60
Released cultivars	5.58	3.90
Farmers cultivars	6.88	2.06
Tuber eye depth		
Breeding clones	6.05	0.90
Released cultivars	6.31	0.94
Farmers cultivars	4.64	2.52
Uniformity of tuber shape		
Breeding clones	5.06	2.08
Released cultivar	4.85	1.66
Farmers cultivars	4.24	4.51

### Genetic structure

**Fig. 1:**
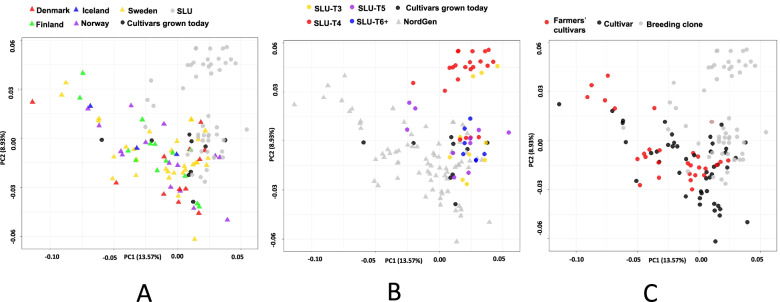
Genetic diversity revealed by a principal component analysis (PCoA) based on Nei’s genetic distance of single nucleotide polymorphism (SNP) markers among the 133 accessions. Groupings are based on (**A)** population and country of origin for NordGen genotypes, (**B)** population and cycle of selection (T3 – T6 +) for SLU breeding clones, and (**C)** clonal type

**Fig. 2:**
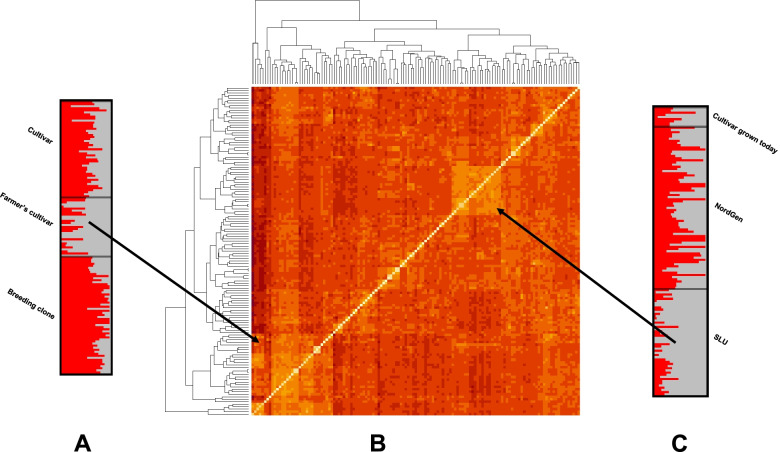
Population structure based on single nucleotide polymorphism (SNP) markers revealed by STRUCTURE (**A** and **C**) and heatmap with dendrogram showing the genetic diversity based on Nei’s genetic (**B**) distance among the 133 accessions. The proposed number of subpopulations (K) was determined according to STRUCTURE as 2 (grey or red), while the assumed number of subpopulations was 3 – cultivar, farmer’s cultivar and breeding clone (**A**); or cultivar grown today in Sweden, NordGen and SLU (**C**). The arrows to the heatmap (**B**) denotes the clusters corresponding to the subpopulations (majority of grey individuals in each biological grouping) proposed by STRUCUTRE

After marker reduction, 11,610 SNP markers remained for all 133 genotypes (Additional File 1). Nei’s genetic distance was used to do the principal coordinate analysis (PCoA) among all genotypes (Fig. 1). A weak population structure was revealed by the PCoA, with a smaller cluster of SLU breeding clones from early clonal generations (T_3_ and T_4_) separating from the remnant accessions (Fig. 1B). The PCoA revealed no clear population structure based on country of origin for the obsolete cultivars held at NordGen (Fig. 1A), or breeding clone generation (Fig. 1C). A heatmap based on Nei’s genetic distance between genotypes also revealed a similar pattern as the PCoA, with a grouping of early selection breeding clones from SLU having a smaller group than remaining accessions (Fig. 2B). The optimal *K* value related to the population structure was two, using a diploid genotyping model for the software ‘STRUCTURE’ [44]. Most genotypes were an admixture of the two populations, suggesting a weak population structure (Fig. 2A and C). However, two clusters in the heatmap did correspond to the two subpopulations proposed by the results from ‘STRUCTURE’, a subset containing predominantly farmer’s cultivars, and a subset containing breeding material from SLU.

### Genetic diversity of potatoes from NordGen

**Fig. 3:**
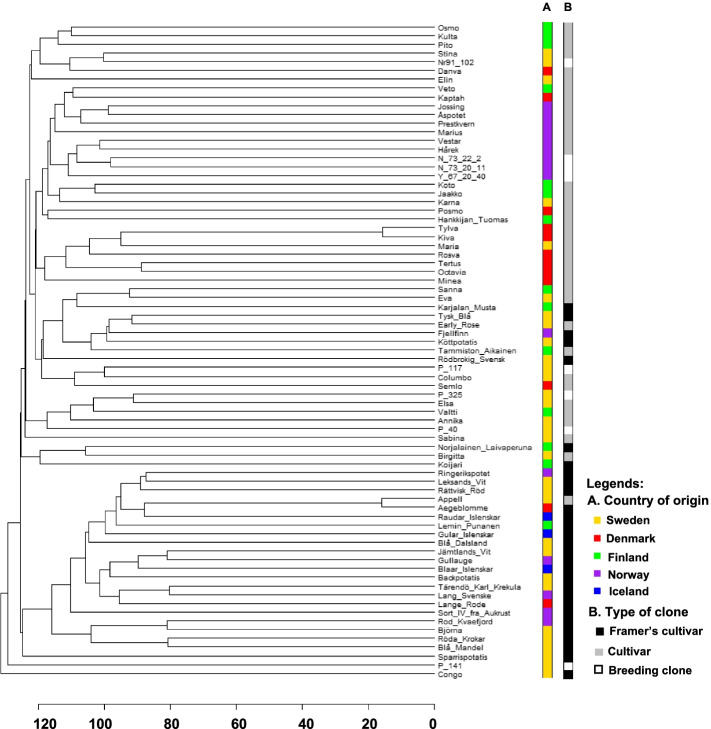
Dendrogram based on the Euclidian distance of single nucleotide polymorphism (SNP) markers among the 75 accessions from NordGen. Groupings made as per country of origin (**A**) or type of clone (**B**)

Using the Euclidian distance from the SNP marker data, a dendrogram was built for the 75 accessions form NordGen (Fig. 3). The 75 accessions were grouped according to both country of origin (Sweden, Denmark, Finland, Norway or Iceland), and type of clone (farmer’s cultivar, released cultivar or breeding clone). A pattern emerged after grouping for type of clone (Fig. 3B): most of the released cultivars and breeding clones formed a distinct group from to the farmer’s cultivars.

A subset of 25 accessions from NordGen was further investigated using another dendrogram, which facilitating comparing it to a previous study using AFLP markers [56]. This dendrogram showed a completely different branching among the subset of accessions (Supplementary Fig. S1) compared to the previously available dendrograms based on either genotypic or phenotypic data. The dendrogram containing the smaller subset of individuals did not display any grouping according to country of origin (Supplementary Fig. S1).

### Level of heterozygosity for 14,730 SNPs

**Fig. 4:**
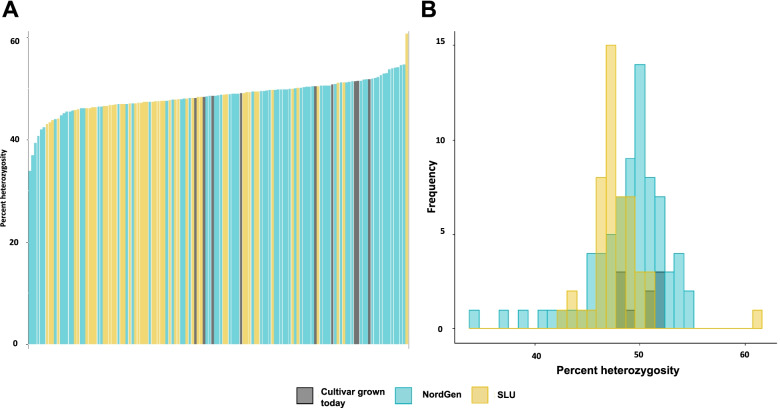
**A** Frequency distribution of percent heterozygosity among all 133 accessions and **B** histogram of percent heterozygosity among the three populations

The level of heterozygosity was defined as the number of heterozygote allele callings in 14,730 SNP markers and was determined for all genotypes. Percent heterozygosity did not differ much among accessions, with a few exceptions. A trend of heterozygosity among accessions from the three groups based on source of the germplasm (NordGen, SLU and grown cultivar) was not observed, neither when studying the genotypes separately (Fig. 4A) or by group (Fig. 4B). The percent heterozygosity was overall lower in SLU breeding clones than in NordGen accessions. However, the mean level of heterozygosity was around 48 to 50% for all three groups based on source of the germplasm.

**Fig. 5:**
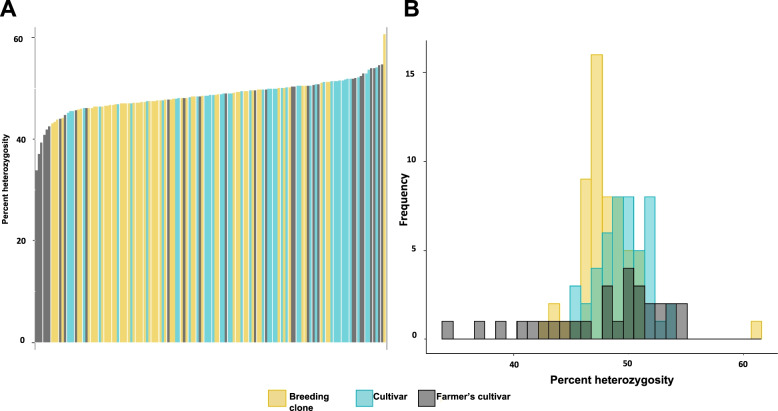
**A** Distribution of percent heterozygosity among all 133 accessions and **B** histogram of percent heterozygosity among the three types of clones

The percent heterozygosity among genotypes when grouped by clonal type followed the same trend and did not show any difference between the groups (Fig. 5A). Farmer’s cultivars had the greatest variation for heterozygosity, and the breeding clones did show a lower percent heterozygosity than released cultivars (Fig. 5B). Nonetheless, the mean level of heterozygosity was very similar between the three clonal types (48–49%).

**Fig. 6:**
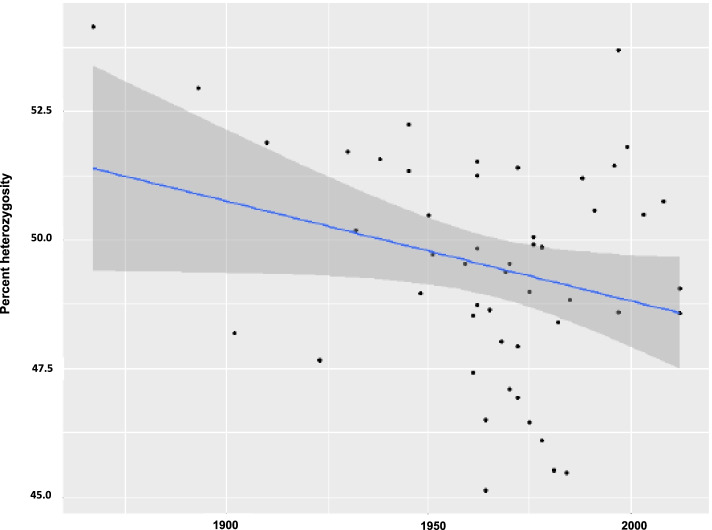
Percent heterozygosity over release year for each of the 51 cultivars with release year information. The negative trend (illustrated by the regression line) is non-significant

The level of heterozygosity was checked over time using available information of year of release for 51 cultivars. Pearson’s product-moment correlation showed a slight but not significant (*P* > 0.05) negative trend (-0.27) towards lower levels of heterozygosity over time (Fig. 6).

## Discussion

Understanding the genetic diversity of breeding germplasm is one of the fundamental tools for a plant breeder, to make informed crosses for the development of new and improved cultivars. By understanding the genetic diversity of *in vitro* collections of potato accessions in genebanks, plant breeders may utilize it wisely in their crossing schemes. In this study, we used SNPs from an array containing 22 K SNP markers to assess the genetic diversity of accessions used for breeding table potatoes for the Swedish market. The accessions included breeding clones from the SLU breeding program; germplasm from NordGen made up of old breeding clones, obsolete cultivars and farmer’s cultivars; and a few cultivars grown today often used as breeding parents in the SLU breeding program.

The number of genotypes (population size) are unevenly distributed among the groups being compared throughout all analysis in this study. The largest difference in population size was found when comparing the three groups based on source of germplasm (NordGen, SLU and cultivars grown today), the group cultivars grown today contain only nine accessions (five with phenotypic data avaliable), and NordGen contain 75 accessions. There are about 25 cultivars commonly grown for the potato market today in Sweden (personal communication: Anders Andersson, Potatisodlarna). Among these 25 cultivars are ‘King Edward’, ‘Bintje, ‘Connect’ and ‘Carolus’, which we have included in this study. The most commonly grown early maturing cultivar in Sweden by far is ‘Solist’. Both the phenotypic and genotypic diversity could be limited to the number of individuals, which must be considered when discussing the results from this study. The grouping based on clonal type has a more even distribution of population size, spanning from 29 (farmer’s cultivars) to 57 (breeding clones) genotypes.

The phenotypic data from the NordGen accesssions has been extensively described in a previous publication [56]. We combined these old phenotypic records with new data recorded for clones from the SLU breeding program to compare the phenotypic diversity among both germplasm sources. Phenotypic traits such as percent dry matter are affected by environmental factors, for example post-harvest storage conditions and soil type [49, 60]. There were no cultivars used as checks in common for the study by Veteläinen et al. [56] and the field trial described here, hence it was not possible to determine any environmental effects on the three phenotypic traits. The results show that on the group level, all measured phenotypic traits differ among the three groups based on source of the germplasm (Table 1). Tuber shape uniformity significantly differs among the groups, and the highest variation for this trait was noted for SLU accessions, and more specifically, among the breeding clones. An explanation to why the genotypes from SLU exhibits a large variation in tuber shape uniformity may be that this trait is not considered as important compared to other breeding traits during the early selection cycles of the breeding program (T_1_-T_4_). A larger proportion of the genotypes from NordGen has a higher percent dry matter content compared to the other two groups, a character that is correlated to mealy potato tubers [8]. When studying the phenotypic variation among the different groups based on clonal type (Table 2), it becomes clear that the higher scores for percent dry matter from NordGen originated from the farmer’s cultivars within this population. The farmer’s cultivars have, on average, higher percent dry matter than the breeding clones and cultivars grown today in Sweden. The largest spread of percent dry matter scores was noticed among the breeding clones. This result suggests that this trait was not the target when making selections in the potato breeding program. The farmer’s cultivars also differ from the two other clonal types regarding tuber eye depth. The farmer’s cultivars seem to have much deeper tuber eyes compared to the two other clonal types. Potato breeders are often selecting for shallow eyes as this makes tubers easier to peel for the consumers of table potato. These farmer’s cultivars did not result from breeding efforts, but instead they reflect what characters potato growers and consumers have favoured. Hence, the tuber characters favoured by end users differ geographically and over time. In Sweden today, ‘King Edward’ remains the favourite table potato mainly due to its appreciated flavour and good properties for the production of mashed potatoes as well for other uses in gastronomy. Such a finding shows that flavour and possibility to use a cultivar for many purposes should be considered when breeding new cultivars.

In general, potato exhibits a low degree of population structure [13, 37], with European cultivars in particular stemming from a very narrow genetic base [18, 52]. The results from our study suggest that there is a very limited population structure among potato cultivars and breeding clones in the Nordic region. In line with what previously was reported by Veteläinen et al. [56], no population structure was observed based on country of origin (Fig. 1A). The NordGen definition of country of origin is, however, limited to and defined as the country in which the accession was collected, and it is known that several of the farmer’s cultivars have been grown in more than a single Nordic country. This as well as the limited genetic base of European potato cultivars in general may have affected the lack of structure based on country of origin (Fig. 1A).

Population structure was examined using a PCoA based on Nei’s pairwise distance among genotypes (Fig. 1A-C) and STRUCTURE [44] with *K* ranging from 0 to 10 (Fig. 2A and C). The *K* with the maximum likelihood was 2, thus suggesting two subpopulations in the data. This theoretical number of subpopulations is to a limited extent explained in the results from the PCoA and heatmap. A group of breeding clones from SLU does cluster separately from the other material. When studying this group more closely, it appears that clones from the early cycles of selection in SLU breeding program are represented in this outlying group (Figs. 1B, 2C). Breeding clones from SLU which have undergone a larger number of cycles of selection are in the same cluster with the rest of the material, thereby suggesting that the potato breeders are actively making selections towards clones that are similar to cultivars grown in Sweden today and farmer’s cultivars previously grown in the Nordic region.

The bar plot from the STRUCTURE output suggests a possible subpopulation division based on farmer’s cultivars versus the remaining materials (Fig. 2A). This division of subpopulations is unclear when studying the PCoA (Fig. 1C). However, there is a cluster represented by farmer’s cultivars appearing in the heatmap (Fig. 2B), which is based on the same genetic data as the PCoA. A similar population structure, where farmer’s cultivars grouped in a separate subpopulation was found in Chinese potato collections [58]. It is still important to keep in mind that almost every genotype was an admixture of both theoretical subpopulations, which suggests a weak population structure.

No structure based on country of origin was observed, which could be explained by the narrow genetic base of the sampled accessions. The pedigree information is limited for potato in general, but it is not inconceivable that the farmer’s cultivars included in this study would appear in the pedigree of the breeding clones either from SLU or among the NordGen accessions. Several of the cultivars grown in Sweden today are actively used as breeding parents at the SLU potato breeding program. It would have been interesting to include genotypes with a wider geographical background, especially outside Europe to get an estimation if the Nordic region has been uniquely differentiated compared to potato grown elsewhere in the world.

Previous research found that market class was a good biological explanation to subpopulations in cultivated tetraploid potato [24, 27, 40]. In this study, all potato accessions are classified as table potato with one exception, the cultivar ‘Kuras’ from the population of cultivars grown in Sweden today. ‘Kuras’ is grown for starch production [3]. We did not, however, observe this as an outlier in our genetic structure analysis, but instead it grouped close to the other Dutch cultivars in the study (data not shown).

The genetic diversity of the potato germplasm from NordGen has been examined previously by Veteläinen et al. [56]. Their study, focusing on finding morphological characters to identify duplicates among accession, included a fraction of the accessions used in our study. All of them were farmer’s cultivars. Our results using a 22 K SNP array supports what Veteläinen et al. [56] noticed, i.e., duplicated accessions of potato did not exist in the NordGen genebank. The study also included a genetic diversity study assessment using 63 AFLPs. The dendrogram from their study based on AFLPs was different than ours based on SNPs (Supplementary Fig. S1). The dendrogram by Veteläinen et al. [56] based on their 57 morphological characters did not match with the dendrogram from our study (Supplementary Fig. S1). A separate dendrogram containing all accessions from the NordGen population was also drawn using the SNP data (Fig. 3). As in the PCoA (Fig. 1A), no population structure was revealed based on country of origin from the dendrogram. When the accessions were assigned as type of clone, the structure could be explained by the clustering in the dendrogram to a certain degree. Two subgroups were revealed, where most of the released cultivars and breeding clones branched separately from the farmer’s cultivars.

Crop yield potential has successfully been improved through heterosis for other crops [7, 9, 29]. It has been theorized that over time, potato breeding will increase the level of heterozygosity due to heterosis related to increased tuber yield [24, 32]. In this regard, Bonierbale et al. [6] found that homozygosity was negative correlated with total tuber yield and yield of tubers of big size in offspring derived from crossing adapted breeding clones. In line with what was found by Hirsch et al. [24] studying US potato cultivars, the level of heterozygosity did not change significantly over time in our study. However, the number of cultivars were significantly larger in the later years compared to the early years, making the estimations skewed.

The average percent heterozygosity ranged from 48 to 50% for each population included in this study. This is a bit lower than what was recorded previously in other germplasms. The average percent heterozygosity was 56% or 57% in germplasm from the USA or Japan respectively [24, 27]. Another US germplasm set was investigated by Pandey et al. [40] with an average percent heterozygosity of 60%. In both assessments in the USA as well as in the study in Japan, a very similar or identical SNP array was utilized for genotyping as in our study. Hence, the genetic variability of the Nordic germplasm is slightly lower. The percent heterozygosity did not vary between populations or types of clones, thus contradicting the theory that breeding would increase the germplasm’s level of heterozygosity, which may otherwise suggest limited genetic gains for tuber yield in potato breeding as already noted in the USA [14].

Employing SNP arrays for genotyping may lead to ascertainment bias in population research such as ours [1, 16, 23, 35]. SNP arrays often show an underrepresentation of SNPs with extreme allele frequency and are limited to the heterozygosity of the loci found using a limited panel of genotypes included in the development of the array. The 22 K SNP array used in this study was developed using 569 unique accessions selected from all over the world, but with an emphasis on European cultivars [57]. Several of the cultivars grown in Sweden today were included in the development of the SNP array (‘Bintje’, ‘Kuras’, ‘Sarpo Mira’, ‘Desirée’ and ‘Bionica’), and some of these cultivars are used as parents in the SLU breeding program. However, only one non-Nordic accession kept at NordGen was included when the array was developed (the US cultivar ‘Early Rose’). Hence, a lot of the genetic diversity present in this group may be unavailable when using this genotyping method.

## Conclusion

The germplasm from NordGen and the potato breeding program at SLU seems to be closely related. There is a slightly larger variation spread among the breeding clones from SLU according to our genetic analyses. While the spread of phenotypes might be larger among the farmer’s cultivars kept at NordGen. The results generated will be of interest to potato breeders in Sweden and other countries of the Nordic region as they consider introducing accessions from NordGen to expand the genetic diversity in their breeding programs.

## Materials and methods

### Plant material

In total, the sample of our study included 133 genotypes from the germplasm collections at the potato-breeding program at SLU (*n* = 49), NordGen (*n* = 75), and a selection of nine released cultivars popularly grown in Sweden today and commonly used as parents for the breeding germplasm at SLU (Supplementary Table S1). From SLU, breeding clones that had undergone at least two cycles of selection (T_3_) were selected, and clonal generation were defined for each genotype until five cycles of selection (T_6+_). The information about the nine released cultivars regarding both country of origin (breeder or breeding company) and release year were available through the Potato Pedigree Database [4, 26]. The accessions from NordGen contained genotypes of three different clonal types: breeding clones (*n* = 8), cultivars (*n* = 38) and farmer’s cultivars (*n* = 29). The origin countries of the accessions from NordGen were defined as the Nordic country in which the genebank did the collecting. Regarding cultivars, there are two, ‘Early Rose’ and ‘Marius’ that are non-Nordic (the former released in the USA and the other in Poland) but in the database they are assigned to Sweden and Norway because of their former extensive cultivation in both.

### Phenotypic data

Phenotypic information was available for 109 of the total 133 genotypes (Additional File 2). Three breeding traits on potato tubers were investigated – percent dry matter content, tuber eye depth, and tuber shape uniformity. Phenotypic information was lacking for two of the released cultivars, and for seven of SLU breeding clones. From the NordGen collection, 60 out of 75 genotypes had phenotypic data available, recorded as described by Veteläinen et al. [56]. The phenotypic data for the genotypes belonging to SLU and cultivars grown today were taken from 20–plant plots grown at three field sites in Sweden (Helgegården 56°02’N 14°07’E, Mosslunda 55°98’N 14°10’E and Umeå 63°84’N 20°26’E) during 2018. Tubers were sown on 14–15^th^ May and harvested on 11–12^th^ October. Tuber shape uniformity was recorded using a scale ranging from 1 (non-uniform) to 9 (uniform), while tuber eye depth was recorded on a scale ranging from 1 (deep) to 9 (shallow) following Selga et al. [50]. Specific gravity was estimated as tuber weight in air divided by subtraction of tuber weight in air and tuber weight in water. The estimates on specific gravity were converted to percent dry matter content as described by Mosley and Chase [33] and adapted to a scale from 1 (low percent dry matter content) to 9 (high percent dry matter content). These 1–9 scales are used by potato genebanks for these characteristics following the descriptor list for the crop [25].

To detect variation of the means of each of the groups – either by source of genotypes (i.e., NordGen, SLU or grown cultivar) or type of clone (i.e., breeding clone, released cultivar or farmer’s cultivar), an analysis of variance was conducted for each of the three breeding traits. If the analysis of variance was significant (*P* < 0.05), Tukey’s range test [54] was conducted to determine which of the three groups differed from one another.

### SNP genotyping

From each of the 133 genotypes, approximately 0.25 g of leaf tissue was sampled for SNP genotyping. The leaf material was collected on ice and kept at -80 °C until shipping to SGS – TraitGenetics GmbH (Gatersleben, Germany) for DNA extraction, genotyping and allele calling. The GGPv3.0 array [57] was used for genotyping. For genotype calling, the software ‘Illumina GenomeStudio’ (Illumina, San Diego, CA) was utilized, scoring four alleles per locus. To ensure the quality of the SNPs to our data set, SNPs with over 10% missing rate were discarded, leaving 14,370 SNPs. Genotype callings were translated from base format (ATCG) to numeric format and missing values were imputed using the function *read.GWASpoly* from the R package ‘GWASpoly’ [48]. The imputation method deployed was population mode, where the most frequent allelic state is used to impute missing marker genotypes. Monomorphic markers and markers with a minor allele frequency below 0.05 were discarded, leaving 11,610 SNPs to be used in further analysis. The R statistical program was used for analysis [45].

### Genotypic data analysis

Population structure was investigated using the set of 11 610 SNPs. The allele format was converted from numeric format (0–4) to an AB format, where A represents the major allele and B represents the minor allele at each given locus. The function *stamppConvert* from the R package ‘StAMPP’ [42] was used to calculate the allele frequency at each genotype. The genetic distance between individuals was calculated based on Nei’s distance [34] using the function *stamppNeisD* from the same R package. To investigate population structure, the genetic distance matrix was displayed in two ways. A heatmap was used to visualise the genetic kinship among the genotypes based on Nei’s genetic distance. In addition to the heatmap, a principal coordinate analysis (PCoA) [19] was carried out to determine the singular value decomposition. The PCoA was carried out using the function *pcoa* from the R package ‘ape’ [41] without correction for negative Eigenvalues. The PCoA was visualized as a scatter plot using the R package ‘ggplot2’ [59].

The genetic relationships among the genotypes from the NordGen population were further investigated with a hierarchical clustering analysis. The Euclidian distance was calculated among the 75 genotypes from NordGen using 11 610 SNPs in numeric format (0–4). Subsequently, a neighbour joining dendrogram was generated using the function *set* from the R package ‘dendexted’ [15]. The country of origin and clonal type for each genotype was visualized using the function *colored_bars* from the same package.

The population structure for all genotypes (*n* = 133) was also determined using the ‘STRUCTURE’ software [44] with an admixture model. As ‘STRUCTURE’ is not developed for tetraploid species, the genotypic data was “diploidized” where the three heterozygote states (AAAB, AABB, and ABBB) where converted to a single heterozygote (AB). Three replicates were performed for each suggested number of populations (*K*) ranging from 1 to 10 with 50 000 replicates of the Markov Chain Monte Carlo after 15 000 burn-ins. The optimal *K* value was determined as the log-likelihood of *K,* and visualized using the ‘STRUCTURE HARVESTER’ web application [10]. The independent outputs from the 3 replicates of the optimal *K* were permuted using the software ‘CLUMPP’ [28] based on the algorithm *FullSearch*. The output was subsequently visualised using the software ‘DISTRUCT’ [47].

The level of heterozygosity of 14,730 SNPs was measured within each genotype as the percent of the heterozygotes SNPs using a diploid model where the three tetraploid heterozygote states were scored as one. The level of heterozygosity over time was estimated using cultivar release year for the 51 cultivars where these data were available. Pearson’s correlation coefficient was calculated to establish any association between release year and level of heterozygosity.

## Supplementary Information

**Additional file 1.**

**Additional file 2.**

**Additional file 3:** **Table S1. **List of the 133 accessions included in the study including information of population (germplasm origin) and clonal type. *Origin for the NordGen population means Nordic country the genotype was collected from, not country of breeding program. **Breeding cycle for breeding clones from the SLU breeding program, T_n_. Where n denotes the number of cycles of selections in the breeding program.

**Additional file 4:** **Figure S1.** The 25 NordGen accessions included in Veteläinen et al. (2005), dendrogram based on our 15 000 single nucleotide polymorphism (SNP) markers.

## Data Availability

The datasets used and analysed during the current study are available in supplementary Additional file 1.csv and Additional file 2.csv for single nucleotide polymorphisms and tuber characteristics, respectively. The Nordic potato cultivars are available from NordGen through requests to be sent directly to PC (pawel.chrominski@nordgen.org).

## Abbreviations

AFLP: Amplified fragment length polymorphism
K: Suggested number of populations
NordGen: Nordic Genetic Resources Center
PCoA: Principal coordinate analysis
SLU: Swedish University of Agricultural Sciences
SNP: Single nucleotide polymorphism
