# Supplementary figures and images for: Diversity and population structure of Nordic potato cultivars and breeding clones
Source: BMC Plant Biol. 2022 Jul 18;22:350. doi: 10.1186/s12870-022-03726-2 (PMC9290215; doi:10.1186/s12870-022-03726-2)

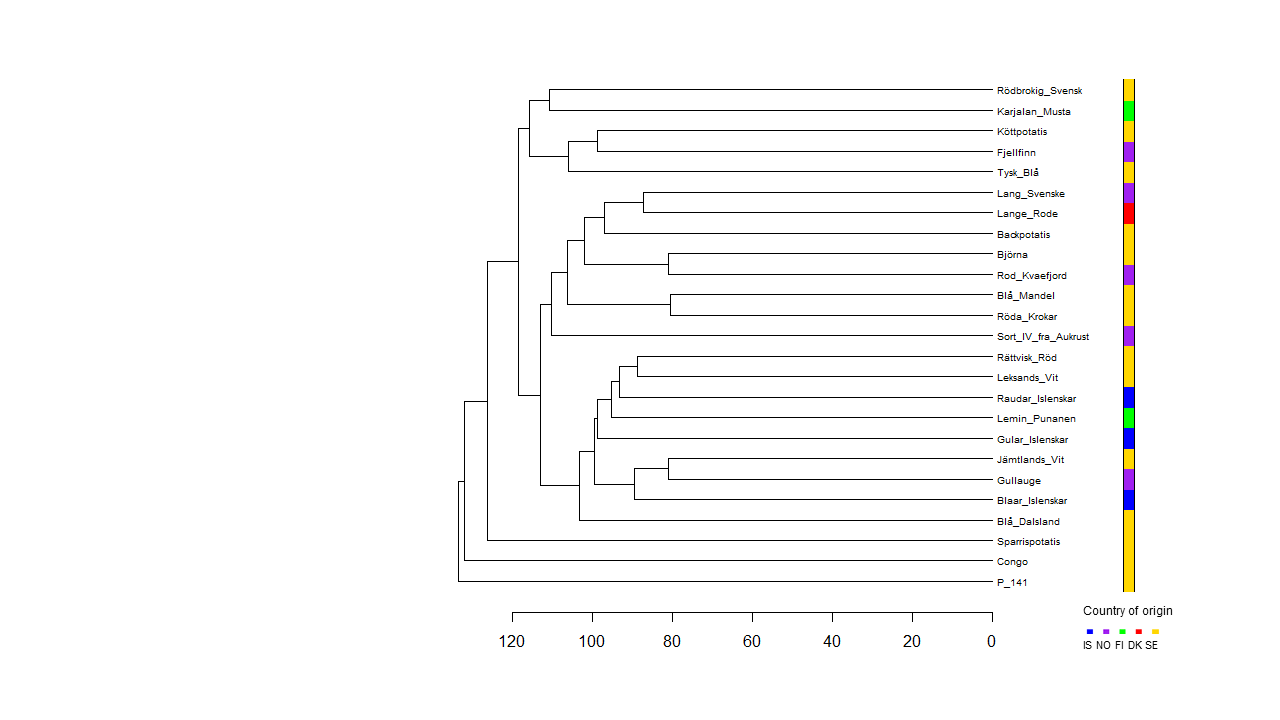

Supplement: Supplementary file 4 — Additional file 4: Figure S1. The 25 NordGen accessions included in Veteläinen et al. (2005), dendrogram based on our 15 000 single nucleotide polymorphism (SNP) markers. [file 12870_2022_3726_MOESM4_ESM.tif]
